# Rifampicin and isoniazid resistance not promote fluoroquinolone resistance in *Mycobacterium smegmatis*

**DOI:** 10.1371/journal.pone.0315512

**Published:** 2025-01-02

**Authors:** Qin Zhou, Na Pu, Ge Xu, Hangchi Liu, Xudong Jia, Xiaomin Wang, Peng Xu

**Affiliations:** 1 National Clinical Research Center for Infectious Diseases, Shenzhen Clinical Research Center for Tuberculosis, Shenzhen Third People’s Hospital, Shenzhen, Guangdong, China; 2 Zunyi Medical University, Zunyi, Guizhou, China; Abdul Wali Khan University Mardan, PAKISTAN

## Abstract

**Background:**

The emergence of drug-resistant Tuberculosis (TB) has made treatment challenging. Although fluoroquinolones (FQs) are used as key drugs in the treatment of multidrug-resistant tuberculosis (MDR-TB), the problem of FQs resistance is becoming increasingly serious. Rifampicin (RIF) resistance is considered a risk factor for FQs resistance. The objective of this study was to investigate the impact of RIF and isoniazid (INH) resistance on the FQs resistance in vitro experiment.

**Methods:**

FQs resistant strains were selected in vitro from RIF and/or INH resistant *Mycobacterium smegmatis* (*M*.*sm*). The sequencing of the *gyrA* gene, and the minimum inhibitory concentration (MIC) of FQs (ciprofloxacin, levofloxacin, moxifloxacin and gatifloxacin) were performed for FQs-resistant strains.

**Results:**

A total of 222 FQs-resistant *M*.*sm* strains were selected, all of which had the *gyrA* mutation. Seven *gyrA* mutations were detected, with mutations at loci 90 and 94 being the most common. There were no differences in FQs resistance developed from RIF and/or INH resistant *M*.*sm*. There was a significant difference in the MIC of the *gyrA* mutant types to FQs. The highest resistance to FQs was observed in the Gly88Cys mutant strains. *M*.*sm* with the identical *gyrA* mutation showed the highest resistance to ciprofloxacin and relatively low resistance to gatifloxacin and moxifloxacin.

**Conclusions:**

In this study, we found no evidence that RIF and/or INH resistance directly affects FQs resistance in *M*.*sm* in vitro experiments. Resistance profiles of different *gryA* mutations to the four FQs drugs were also presented. These findings provide a more comprehensive understanding of FQs resistance.

## Introduction

Tuberculosis (TB) is one of the three major infectious diseases threatening global public health, and is caused by infection with *Mycobacterium tuberculosis* (*M*.*tb*) [1]. The WHO reports that 10.6 million people worldwide had TB in 2022, an increase of 300,000 compared to 2021. Of these, 7.5 million were newly diagnosed with TB, the highest peak in 28 years of global TB surveillance[2, 3]. Approximately 500,000 multidrug-resistant/rifampicin-resistant TB (MDR/RR-TB) cases are reported each year, and drug-resistant is a major cause of TB deaths [4]. The cure rate for MDR/RR-TB patients is only 63%, posing a serious threat to public health [5–8].

Fluoroquinolones (FQs) are important drugs in the treatment of MDR/RR-TB [9]. FQs are commonly used in the treatment of drug-resistant TB because of their efficacy, oral administration, relatively few side effects and low cost compared with other second-line injectable drugs [10]. FQs can inhibit bacterial DNA gyrase and disrupt genome replication to exert bactericidal activity. Bacteria develop resistance to FQs through mutations in the *gyrA* gene, which encodes a DNA gyrase [11, 12]. In recent years, FQs resistant TB has become increasingly common in clinics, posing a challenge to treating the disease. Treatment success rates for pre-XDR (resistant to one of the FQs while fulfil the definition of MDR/RR-TB) and XDR (resistant to bedaquiline or linezolid while fulfil the definition of pre-XDR) TB are only 30~40% [5, 13–15].

Clinical data show that the drug resistance profile may influence the risk of other drug resistance in TB. Compared to susceptible TB, rifampicin (RIF) monoresistant patients and MDR patients had a 6.3-fold and 13.8-fold risk of FQs resistance, respectively [16, 17]. Therefore, to guide the use of FQs in the clinic, it is essential to clarify whether the drug-resistant background influences the development of resistance to FQs. Due to the slow growth and biosafety concerns of *M*.*tb*, *Mycobacterium smegmatis* (*M*.*sm*) is often used as a model organism to study *M*.*tb* drug resistance [18–22]. Therefore, we used *M*.*sm* to investigate whether RIF and/or isoniazid (INH) resistance influences the development of FQs resistance.

## Materials and methods

### RIF-R, INH-R and MDR strains

To investigate the effect of different drug-resistant *M*.*sm* on FQ resistance, the RIF-resistant (RIF-R), INH-resistant (INH-R), MDR (both RIF and INH resistant), and drug-susceptible (both RIF and INH susceptible) MC^2^-155 strains were used, which were *rpoB* gene S531L mutant (MS^RIF-R^), *katG* gene G280D mutant (MS^INH-R^), *rpoB* gene S531L and *katG* gene G280D double mutant (MS^MDR^), and wild type (MS^S^), respectively.

### In vitro selection of FQs-resistant mutants

We isolated spontaneous mutant colonies using 2× minimal inhibitory concentration (MIC) [23] of levofloxacin (0.5 μg/mL) with the Luria-Delbrück fluctuation assay [24]. For the initial preparation of the cell suspension, the optical density (OD) at 600 nm was used to measure the CFU, as a bacterial solution with an OD600 equal to 1.0 corresponds to approximately 2×10^8^ CFU/ml [25]. For each strain, an approximately 2.3 × 105 CFU were added to 90 ml of Middlebrook 7H9 liquid medium containing 10% OADC (7H9-OADC), mixed well, and divided into 20 culture tubes. Each tube contains 4ml of cell suspension, approximately 104 CFU. These parallel cultures were then incubated at 37°C until the OD_600_ reached 1.0 [26]. Among twenty parallel cultures for each strain, 15 were used for isolating spontaneous FQ-resistant mutant colonies and 5 for counting CFU. Spontaneous FQ-resistant mutant colonies were selected on Middlebrook 7H11 solid medium with 10% OADC (7H11-OADC) and 1.0 μg/mL levofloxacin (2× MIC). Serially diluted cell suspensions were added to drug-free 7H11-OADC for CFU counting. Mutation rates and 95% confidence intervals (CI) were calculated by the online tools of FluCalc, which was able to accurately estimate mutation rates using the fluctuation assay [27].

### DNA extraction and *gyrA* gene sequencing

Bacterial genomic DNA was extracted by boiling method [28, 29]. The gyrA-1R (5’-CGACGAACTGTTCTCCATCC-3’) and gyrA-1F (5’-CCGGTCTTGTACGTGTCCTC-3’) primers were used to amplify the 869 bp fragment of the *gyrA* gene [13]. The obtained PCR products were sent for Sanger sequencing to identify the FQs-resistant mutations.

### Minimal inhibitory concentration (MIC) assay for FQs

MICs were determined in 7H9-OADC by the resazurin microtiter assay (REMA) using a twofold FQ drugs dilution [30, 31]. Ciprofloxacin was tested at a range of 0.25 to 128 μg/mL, while levofloxacin at a range of 0.0156 to 8 μg/mL, moxifloxacin at a range of 0.0625 to 32 μg/mL and gatifloxacin at a range of 0.0625 to 32 μg/mL. The MIC is interpreted as the minimum drug concentration that prevents the color changing from blue to purple or pink.

### Statistical analysis

The chi-squared test was used to compare the mutations between the groups, while the one-way ANOVA method and the *t*-test were used to compare the MIC. All analyses were performed with SPSS version 29.0 and results with a *p*-value less than 0.05 were considered statistically significant.

## Results

### Selection of FQs-resistant clones

A total of 609 FQs-resistant clones were selected by the Luria-Delbrück fluctuation assay. The number of FQs-resistant clones from MS^S^, MS^INH-R^, MS^RIF-R^ and MS^MDR^ were 96, 349, 88 and 76, respectively. The frequency of FQs resistance was 2.93×10^−10^ (95% CI: 1.67–4.42×10^−10^) for MS^S^, 5.28×10^−10^ (95% CI: 3.17–7.77×10^−10^) for MS^INH-R^, 8.11×10^−10^ (95% CI: 4.33–12.72×10^−10^) for MS^RIF-R^ and 5.09×10^−10^ (95% CI: 2.56–8.23×10^−10^) for MS^MDR^. Of the 609 FQs-resistant clones, 222 were randomly selected for *gyrA* sequencing and FQs MICs testing, of which 66 clones were from MS^S^, 66 clones from MS^INH-R^, 41 clones from MS^RIF-R^ and 49 clones from MS^MDR^.

### FQs-resistant mutations

Each sequenced clone harbored a mutation in the *gyrA* gene. There were seven types of mutation, located at loci 88, 90, 91 and 94 of the *gyrA* gene (Table 1). Mutations at loci 90 and 94 were the most common, accounting for 54.1% (120/222) and 30.6% (68/222) of all samples, respectively. Chi-squared test showed a significant difference in the mutation profile between MS^S^ and MS^INH-R^ (*p* = 0.004), and between MS^INH-R^ and MS^RIF-R^ (*p* = 0.002) (S1 Table). The mutation profile comparisons between MS^S^ and MS^INH-R^, and between MS^INH-R^ and MS^RIF-R^ were performed independently. Compared to MS^INH-R^, the MS^S^ strain had a significantly higher proportion of Gly88Cys and Asp94Tyr mutations, but a significantly lower proportion of Asp94Gly mutation. And MS^INH-R^ strain had a significantly higher proportion of Ala90Val mutation, but a significantly lower proportion of Ser921Pro mutation than MS^RIF-R^ strain.

**Table 1:** *gyrA* mutation profile of *M*.*sm* strains.

Mutation	MS^S^	MS^INH-R^	MS^RIF-R^	MS^MDR^
	n = 66 (%)	n = 66 (%)	n = 41 (%)	n = 49 (%)
Gly88Cys	11 (16.7)	2 (3.0)	5 (12.2)	3 (6.1)
Ala90Val	36 (54.5)	40 (60.6)	14 (34.1)	30 (61.2)
Ser91Pro	3 (4.5)	1 (1.5)	9 (22.0)	0 (0.0)
Asp94His	0 (0.0)	1 (1.5)	1 (2.4)	0 (0.0)
Asp94Tyr	6 (9.1)	0 (0.0)	0 (0.0)	2 (4.1)
Asp94Gly	10 (15.2)	21 (31.8)	12 (29.3)	14 (28.6)
Asp94Ala	0 (0.0)	1 (1.5)	0 (0.0)	0 (0.0)

Abbreviations: Gly, Glycine; Cys, cysteine; Ala, Alanine; Val, Valine; Ser, Serine; Pro, Proline; Asp, Aspartic acid; His, Histidine; Tyr, Threonine.

### FQs MIC of *M*.*sm* strains

**Fig 1:**
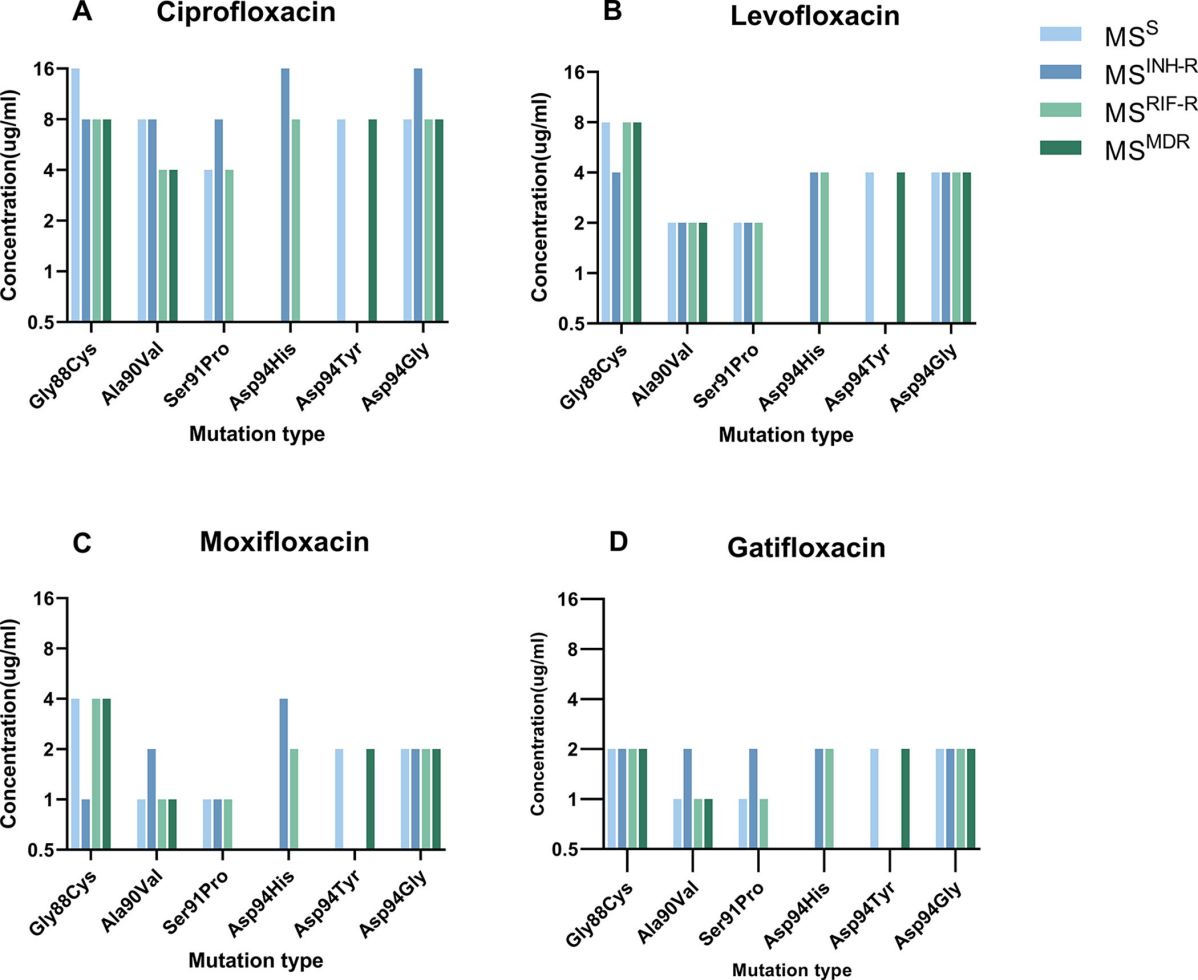
The FQs MIC among *M*.*sm* strains. (A) Ciprofloxacin, (B) Levofloxacin, (C) Moxifloxacin, (D) Gatifloxacin. No clone with *gyrA* Ser91Pro mutation was selected form MS^MDR^, no clone with *gyrA* Asp94His mutation was selected form MS^S^ and MS^MDR^, and no clone with *gyrA* Asp94Tyr mutation was selected form MS^INH-R^ and MS^RIF-R^.

In order to ascertain whether drug-resistance backgrounds influence FQs resistance, this study analyzed the MIC of FQs in MS^S^, MS^INH-R^, MS^RIF-R^, and MS^MDR^ with identical *gyrA* mutations. The MICs of ciprofloxacin, levofloxacin, moxifloxacin and gatifloxacin for strains with the same mutation were found to be generally consistent, with MIC differences observed primarily within a range of one titer (Fig 1). The results indicated that resistance to RIF and/or INH did not influence the level of FQs resistance. Consequently, the MIC results of different strains with the identical mutation were combined and subjected to further analysis. (S2 and S3 Tables).

### FQs MIC of *gyrA* mutations

**Fig 2:**
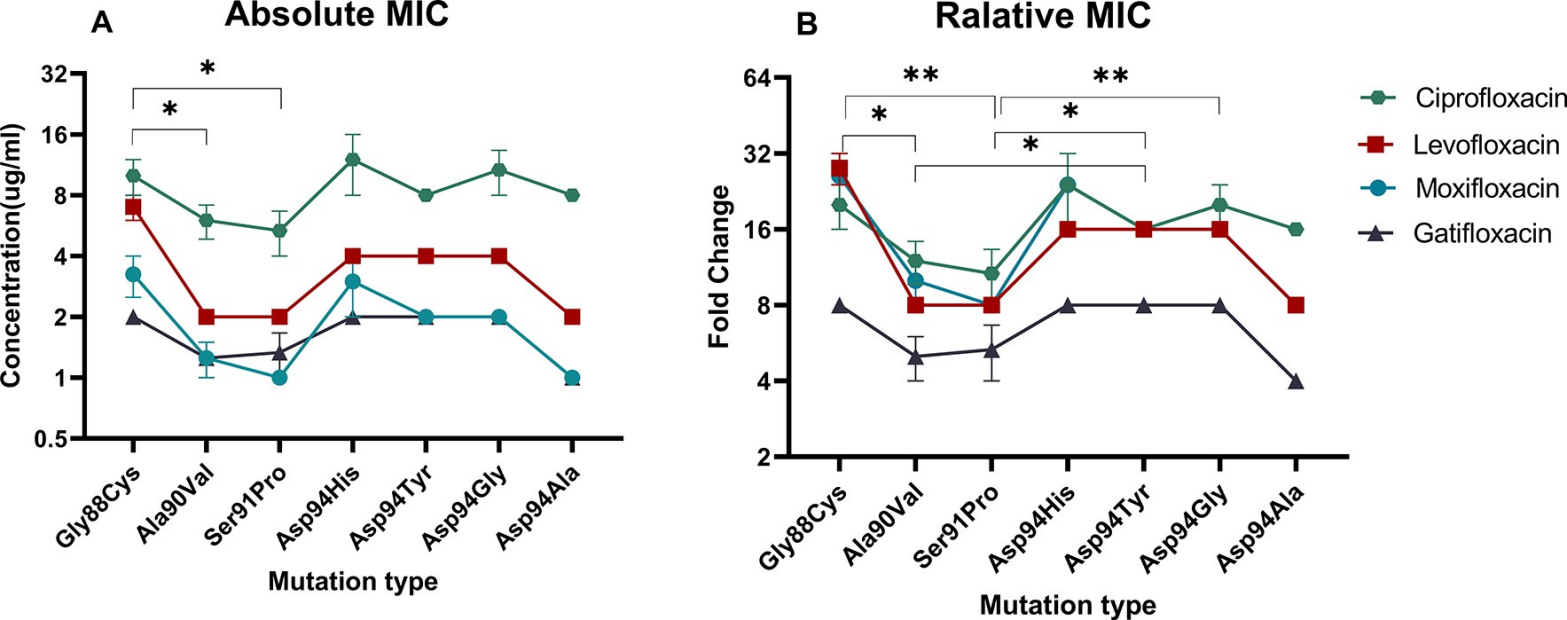
The FQs absolute MIC (A) and relative MIC (B) of *gryA* mutations. * indicates a *p*-value of less than 0.05, ** indicates a *p*-value of less than 0.01.

The *gyrA* mutation increased the MIC of ciprofloxacin from 0.5μg/mL to 4.0~16.0 μg/mL, levofloxacin from 0.25μg/mL to 2.0~8.0 μg/mL, moxifloxacin from 0.125μg/mL to 1.0~4.0 μg/mL, and gatifloxacin from 0.25μg/mL to 1.0~2.0 μg/mL (Fig 1). An analysis of the FQs MICs for different mutations revealed that the MIC of the Gly88Cys mutation was significantly higher than that of the Ala90Val (*p* = 0.015) and Ser91Pro (*p* = 0.016) mutations (Fig 2A). This suggests that the Gly88Cys mutation may confer higher resistance to FQs, while Ala90Val and Ser91Pro confer lower. No significant difference was observed in the MICs between the remaining mutations.

### Differences in MIC of four FQ drugs

In general, the mutant clones exhibited variability in their MICs for the four FQ drugs. The mutant clones had the highest resistance to ciprofloxacin (4.0~16.0 μg/mL), followed by levofloxacin (2.0~8.0μg/mL), and the lowest resistance to moxifloxacin (1.0~4.0 μg/mL) and gatifloxacin (1.0~2.0 μg/mL) (Fig 1). With the exception of no difference being found in MICs between moxifloxacin and gatifloxacin, significant differences were observed in the comparisons of MICs for all the drugs (*p*<0.001) (S4 Table)

The relative changes in the FQs MIC of the mutant strains relative to the wild-type strain were further analyzed. Resistance mutations resulted in the smallest mean increase in minimum inhibitory concentration (MIC) for gatifloxacin, with a mean increase of 6.8-fold (interquartile range: 4–8), whereas levofloxacin, ciprofloxacin, and moxifloxacin showed a larger mean increase in MIC, with a mean increase of 16-fold (IQR: 8–16) (Fig 2B). The relative changes in MIC of gatifloxacin were significantly lower than those of levofloxacin, ciprofloxacin, and moxifloxacin (*p*<0.001) (S5 Table). Among all the mutation types, Ala90Val and Ser91Pro showed lower relative changes than Gly88Cys, Asp94Tyr and Asp94Gly (Fig 2B).

## Discussion

In this study, a total of 222 FQs-resistant *M*.*sm* strains were selected in vitro from RIF and/or INH resistant *M*.*sm*, all of which had the *gyrA* mutation. The MICs of FQs developed from *M*.*sm* strains resistant to RIF and/or INH were found to be no significantly different. Among seven *gyrA* mutations were detected, the highest resistance to FQs was observed in the Gly88Cys mutant strains. *M*.*sm* with the identical *gyrA* mutation showed the highest resistance to ciprofloxacin and relatively low resistance to gatifloxacin and moxifloxacin. The findings will contribute to a more comprehensive understanding of drug resistance in FQs.

The *gyrA* gene encodes the α-subunit of bacterial DNA gyrase, which is the target of FQ drugs [32, 33]. FQ resistance is the consequence of the drug-resistant mutations in the *gyrA* gene [34, 35]. A total of seven FQ-resistant mutations at four codon sites have been observed in this study. All of these mutations have been reported in clinical FQ-resistant *M*.*tb* strains [36–38]. We also found more than one mutation type in codon 94 conferring FQ resistance, which is consistent with clinical *M*.*tb* strains [36, 39]. These results suggest that *M*.*sm* can be used as a model for the study of FQ resistance in *M*.*tb*.

Clinical studies have demonstrated that the prevalence of FQs resistance in MDR/RR-TB cases is considerably higher than in susceptible cases, indicating that INH/RIF resistance may contribute to the risk of FQs resistance [40, 41]. Nevertheless, our study did not identify a significant difference in the frequency of FQs resistance among RIF and/or INH-resistant *M*.*sm*. This suggests that it is not drug resistance profiles but other factors that increase the risk of FQ resistance. One potential explanation is that FQs are primarily used for the treatment of drug-resistant TB [9], whereas patients with susceptible TB have limited opportunity to use FQs. Consequently, the overall FQs resistance rate is higher in drug-resistant TB than in susceptible TB [41]. Studies have shown that subinhibitory concentrations of drugs induce bacteria to generate reactive oxygen species (ROS) that increase the mutation rate [42], thereby increasing the risk of drug resistance. Because the in vivo environment is complex and drug concentrations in tissues vary spatially and temporally [43, 44], it is possible that subinhibitory concentrations may exist in patients and increase the rate of drug resistance mutations. Compared to susceptible strains, drug-resistant strains have more opportunities to be in a subinhibitory environment, which could potentially favour the development of drug resistance.

Not only patient factors, but also bacterial factors other than drug resistance may promote resistance to FQs. Bacteria can prevent drugs from entering into cells to reduce its susceptibility, and can also excrete drugs out of bacteria through efflux pumps to increase the ability of drug resistance, which involving many genes, such as *pe11*, *mymA*, *virS*, *cpnT*, *mmpL3*, *bacA*, *efpA* [45]. These mechanisms that affect drug susceptibility are usually not specific to a single drug, but affect multiple drugs [46]. Drug-resistant strain can also acquire compensatory mutation that partially or fully restore the deficiency caused by the drug resistance conferring mutations [47]. A secondary in the *rpoA*, *ropB* and *rpoC* genes was a common compensatory mutation to restore the deficiency caused by RIF resistance [48], may have a potential contribution to the development of FQs resistance. In addition, genetic background may have an effect on susceptibility to FQs, as the MIC of lineage 3 strains was lower than that of lineages 4 [49]. Whether these bacterial factors contribute to the promotion of FQs resistance requires further investigation.

FQs are frequently used to treat MDR-TB patients as an important component of the WHO-recommended short-course treatment regimen for TB [50, 51]. FQs were initially discovered as broad-spectrum antibiotics in 1962, and four generations of FQ drugs have been developed to date [52]. Our study involved three generations of FQs, including second-generation (ciprofloxacin), third-generation (levofloxacin), and fourth-generation (moxifloxacin and gatifloxacin) [53]. Strains with the same *gyrA* mutation differed in their resistance to different generations of FQs. In general, the more recent generation of FQs exhibited a lower MIC. The MIC of ciprofloxacin for the Gly88Cys mutant strain was 16 μg/mL, while that of gatifloxacin was 2 μg/mL, representing an 8-fold difference. This phenomenon makes it possible for patients to achieve the same therapeutic effect and reduce the risk of side effects by taking lower doses of new-generation FQs. It is also possible to treat low-level drug-resistant TB by using high-dose new-generation FQs.

This study had several limitations. Although, *M*.*sm* is often used as a model organism to study *M*.*tb* drug resistance [18–22], there are genetic and physiological differences between them. The results of this study require further validation using both *M*.*tb* reference and clinical strains with different drug resistance profiles. Secondly, the diversity and complexity of drug resistance mechanisms in clinical *M*.*tb* strains, which may have evolved under different selective pressures in vivo [54], were not considered in this study. Thirdly, this study did not assess the effect of other bacterial factors on the development of resistance to FQs, in particular compensatory mutations, which are common in clinical RIF-resistant strains. These factors need to be studied in order to draw more comprehensive conclusions.

In summary, this study assesses the impact of RIF and/or INH-resistant profile on the development of FQs resistance in *M*.*sm*. We found that there was no significant direct effect of RIF and/or INH resistance profile on FQs resistance in *M*.*sm* in vitro experiments. This indicates that there are other reasons for the higher FQs-resistant rate in drug-resistant TB. Furthermore, the distribution of MICs between generations of FQs and *gyrA* mutations was presented. These findings provide a more comprehensive understanding of FQs resistance.

## Supporting information

S1 Table The original data and *p*-value for between-group differences in mutation composition ratios for different drug-resistant strains. (DOCX)

S2 Table The original data and *p*-value for difference in absolute MIC between groups of different resistant strains. (DOCX)

S3 Table The original data and *p*-values of differences in relative MICs between different strains. (DOCX)

S4 Table The original data and *p*-value for difference in absolute MIC between groups of different FQs. (DOCX)

S5 Table The original data and *p*-value for difference in relative MIC between groups of different FQs. (DOCX)
