# Correction: Rifampicin and isoniazid resistance not promote fluoroquinolone resistance in *Mycobacterium smegmatis*

**DOI:** 10.1371/journal.pone.0338297

**Published:** 2025-12-05

**Authors:** Qin Zhou, Na Pu, Ge Xu, Hangchi Liu, Xudong Jia, Xiaomin Wang, Peng Xu

The authors, Xiaomin Wang and Peng Xu, should be noted as shared co-corresponding authors on this work. Xiaomin Wang’s email address is: WXM_ZMU@163.com.

The corresponding author’s email address is incorrect. Peng Xu’s email address is: PengXu_NCRC@163.com.
