# Supplementary material for: Rifampicin and isoniazid resistance not promote fluoroquinolone resistance in Mycobacterium smegmatis
Source: PLoS One. 2025 Jan 2;20(1):e0315512. doi: 10.1371/journal.pone.0315512 (PMC11694965; doi:10.1371/journal.pone.0315512)
Supplement: S1 Table — (DOCX) [file pone.0315512.s001.docx]

**S1 Table.** The original data and *p*-value for between-group differences in mutation composition ratios for different drug-resistant strains.

| **Mutation** | **MS^S^** | **MS^INH-R^** | **MS^RIF-R^** | **MS^MDR^** |
| --- | --- | --- | --- | --- |
|  | **n=66 (%)** | **n=66 (%)** | **n=41 (%)** | **n=49 (%)** |
| **Gly88Cys** | 11 (16.7) | 2 (3.0) | 5 (12.2) | 3 (6.1) |
| **Ala90Val** | 36 (54.5) | 40 (60.6) | 14 (34.1) | 30 (61.2) |
| **Asp94Gly** | 10 (15.2) | 21 (31.8) | 12 (29.3) | 14 (28.6) |
| **Other** | 9 (13.6) | 3 (4.5) | 10 (24.4) | 2 (4.1) |

| **MS^INH-R^** | 0.004 |  |  |
| --- | --- | --- | --- |
| **MS^RIF-R^** | 0.083 | 0.002 |  |
| **MS^MDR^** | 0.048 | 0.866 | 0.011 |
|  | **MS^S^** | **MS^INH-R^** | **MS^RIF-R^** |
