# Supplementary material for: Rifampicin and isoniazid resistance not promote fluoroquinolone resistance in Mycobacterium smegmatis
Source: PLoS One. 2025 Jan 2;20(1):e0315512. doi: 10.1371/journal.pone.0315512 (PMC11694965; doi:10.1371/journal.pone.0315512)
Supplement: S2 Table — (DOCX) [file pone.0315512.s002.docx]

**S2 Table.** The original data and *p*-value for difference in absolute MIC between groups of different resistant strains.

|  | **MIC（ug/ml）** | | | | | | | | | | | |
| --- | --- | --- | --- | --- | --- | --- | --- | --- | --- | --- | --- | --- |
| **Strain** | **MS^S^** | | | **MS^INH-R^** | | | **MS^RIF-R^** | | | **MS^MDR^** | | |
| **Mutation** | **Gly88Cys** | **Ala90Val** | **Asp94Gly** | **Gly88Cys** | **Ala90Val** | **Asp94Gly** | **Gly88Cys** | **Ala90Val** | **Asp94Gly** | **Gly88Cys** | **Ala90Val** | **Asp94Gly** |
| **Moxifloxacin** | 4 | 1 | 2 | 1 | 2 | 2 | 4 | 1 | 2 | 4 | 1 | 2 |
| **Levofloxacin** | 8 | 2 | 4 | 4 | 2 | 4 | 8 | 2 | 4 | 8 | 2 | 4 |
| **Gatifloxacin** | 2 | 1 | 2 | 2 | 2 | 2 | 2 | 1 | 2 | 2 | 1 | 2 |
| **Ciprofloxacin** | 16 | 8 | 8 | 8 | 8 | 16 | 8 | 4 | 8 | 8 | 4 | 8 |

| **MS^INH-R^** | 0.706 |  |  |
| --- | --- | --- | --- |
| **MS^RIF-R^** | 0.191 | 0.522 |  |
| **MS^MDR^** | 0.191 | 0.522 | * |
|  | **MS^S^** | **MS^INH-R^** | **MS^RIF-R^** |

*Correlation and t could not be calculated because the standard error of the difference between MS^RIF^ and MS^MDR^ bacteria was 0.

**S3 Table.** The original data and *p*-values of differences in relative MICs between different strains.

|  | **Relative MIC** | | | | | | | | | | | |
| --- | --- | --- | --- | --- | --- | --- | --- | --- | --- | --- | --- | --- |
| **Strain** | **MS^S^** | | | **MS^INH-R^** | | | **MS^RIF-R^** | | | **MS^MDR^** | | |
| **Mutation** | **Gly88Cys** | **Ala90Val** | **Asp94Gly** | **Gly88Cys** | **Ala90Val** | **Asp94Gly** | **Gly88Cys** | **Ala90Val** | **Asp94Gly** | **Gly88Cys** | **Ala90Val** | **Asp94Gly** |
| **Moxifloxacin** | 32 | 8 | 16 | 8 | 16 | 16 | 32 | 8 | 16 | 32 | 8 | 16 |
| **Levofloxacin** | 32 | 8 | 16 | 16 | 8 | 16 | 32 | 8 | 16 | 32 | 8 | 16 |
| **Gatifloxacin** | 8 | 4 | 8 | 8 | 8 | 8 | 8 | 4 | 8 | 8 | 4 | 8 |
| **Ciprofloxacin** | 32 | 16 | 16 | 16 | 16 | 32 | 16 | 8 | 16 | 16 | 8 | 16 |

| **MS^INH-R^** | 0.482 |  |  |
| --- | --- | --- | --- |
| **MS^RIF-R^** | 0.191 | 0.915 |  |
| **MS^MDR^** | 0.191 | 0.915 | * |
|  | **MS^S^** | **MS^INH-R^** | **MS^RIF-R^** |

*Correlation and t could not be calculated because the standard error of the difference between MS^RIF^ and MS^MDR^ bacteria was 0.

|  | **MIC（ug/ml）** | | | | | | | | | | | | | | | | | | | |
| --- | --- | --- | --- | --- | --- | --- | --- | --- | --- | --- | --- | --- | --- | --- | --- | --- | --- | --- | --- | --- |
| **Mutation** | **Gly88Cys** | | | | **Ala90Val** | | | | **Ser91Pro** | | | **Asp95His** | | **Asp95Tyr** | | **Asp95Gly** | | | | **Asp95Ala** |
| **Moxifloxacin** | 4 | 1 | 4 | 4 | 1 | 2 | 1 | 1 | 1 | 1 | 1 | 4 | 2 | 2 | 2 | 2 | 2 | 2 | 2 | 1 |
| **Levofloxacin** | 8 | 4 | 8 | 8 | 2 | 2 | 2 | 2 | 2 | 2 | 2 | 4 | 4 | 4 | 4 | 4 | 4 | 4 | 4 | 2 |
| **Gatifloxacin** | 2 | 2 | 2 | 2 | 1 | 2 | 1 | 1 | 1 | 2 | 1 | 2 | 2 | 2 | 2 | 2 | 2 | 2 | 2 | 1 |
| **Ciprofloxacin** | 16 | 8 | 8 | 8 | 8 | 8 | 4 | 4 | 4 | 8 | 4 | 16 | 8 | 8 | 8 | 8 | 16 | 8 | 8 | 8 |

**S4 Table.** The original data and *p*-value for difference in absolute MIC between groups of different FQs.

| **Levofloxacin** | ＜0.001 |  |  |
| --- | --- | --- | --- |
| **Gatifloxacin** | ＜0.001 | ＜0.001 |  |
| **Moxifloxacin** | ＜0.001 | ＜0.001 | 0.163 |
|  | **Ciprofloxacin** | **Levofloxacin** | **Gatifloxacin** |

**S5 Table.** The original data and *p*-value for difference in relative MIC between groups of different FQs.

|  | **Relative MIC** | | | | | | | | | | | | | | | | | | | |
| --- | --- | --- | --- | --- | --- | --- | --- | --- | --- | --- | --- | --- | --- | --- | --- | --- | --- | --- | --- | --- |
| **Mutation** | **Gly88Cys** | | | | **Ala90Val** | | | | **Ser91Pro** | | | **Asp95His** | | **Asp95Tyr** | | **Asp95Gly** | | | | **Asp95Ala** |
| **Moxifloxacin** | 32 | 8 | 32 | 32 | 8 | 16 | 8 | 8 | 8 | 8 | 8 | 32 | 16 | 16 | 16 | 16 | 16 | 16 | 16 | 8 |
| **Levofloxacin** | 32 | 16 | 32 | 32 | 8 | 8 | 8 | 8 | 8 | 8 | 8 | 16 | 16 | 16 | 16 | 16 | 16 | 16 | 16 | 8 |
| **Gatifloxacin** | 8 | 8 | 8 | 8 | 4 | 8 | 4 | 4 | 4 | 8 | 4 | 8 | 8 | 8 | 8 | 8 | 8 | 8 | 8 | 4 |
| **Ciprofloxacin** | 32 | 16 | 16 | 16 | 16 | 16 | 8 | 8 | 8 | 16 | 8 | 32 | 16 | 16 | 16 | 16 | 32 | 16 | 16 | 16 |

| **Levofloxacin** | 0.385 |  |  |
| --- | --- | --- | --- |
| **Gatifloxacin** | ＜0.001 | ＜0.001 |  |
| **Moxifloxacin** | 0.629 | 0.428 | ＜0.001 |
|  | **Ciprofloxacin** | **Levofloxacin** | **Gatifloxacin** |
