# Supplementary material for: Rifampicin and isoniazid resistance not promote fluoroquinolone resistance in Mycobacterium smegmatis
Source: PLoS One. 2025 Jan 2;20(1):e0315512. doi: 10.1371/journal.pone.0315512 (PMC11694965; doi:10.1371/journal.pone.0315512)
Supplement: S3 Table — (DOCX) [file pone.0315512.s003.docx]

**S3 Table.** The original data and *p*-values of differences in relative MICs between different strains.

|  | **Relative MIC** | | | | | | | | | | | |
| --- | --- | --- | --- | --- | --- | --- | --- | --- | --- | --- | --- | --- |
| **Strain** | **MS^S^** | | | **MS^INH-R^** | | | **MS^RIF-R^** | | | **MS^MDR^** | | |
| **Mutation** | **Gly88Cys** | **Ala90Val** | **Asp94Gly** | **Gly88Cys** | **Ala90Val** | **Asp94Gly** | **Gly88Cys** | **Ala90Val** | **Asp94Gly** | **Gly88Cys** | **Ala90Val** | **Asp94Gly** |
| **Moxifloxacin** | 32 | 8 | 16 | 8 | 16 | 16 | 32 | 8 | 16 | 32 | 8 | 16 |
| **Levofloxacin** | 32 | 8 | 16 | 16 | 8 | 16 | 32 | 8 | 16 | 32 | 8 | 16 |
| **Gatifloxacin** | 8 | 4 | 8 | 8 | 8 | 8 | 8 | 4 | 8 | 8 | 4 | 8 |
| **Ciprofloxacin** | 32 | 16 | 16 | 16 | 16 | 32 | 16 | 8 | 16 | 16 | 8 | 16 |

| **MS^INH-R^** | 0.482 |  |  |
| --- | --- | --- | --- |
| **MS^RIF-R^** | 0.191 | 0.915 |  |
| **MS^MDR^** | 0.191 | 0.915 | * |
|  | **MS^S^** | **MS^INH-R^** | **MS^RIF-R^** |

*Correlation and t could not be calculated because the standard error of the difference between MS^RIF^ and MS^MDR^ bacteria was 0.
