# Supplementary material for: Rifampicin and isoniazid resistance not promote fluoroquinolone resistance in Mycobacterium smegmatis
Source: PLoS One. 2025 Jan 2;20(1):e0315512. doi: 10.1371/journal.pone.0315512 (PMC11694965; doi:10.1371/journal.pone.0315512)
Supplement: S5 Table — (DOCX) [file pone.0315512.s005.docx]

**S5 Table.** The original data and *p*-value for difference in relative MIC between groups of different FQs.

|  | **Relative MIC** | | | | | | | | | | | | | | | | | | | |
| --- | --- | --- | --- | --- | --- | --- | --- | --- | --- | --- | --- | --- | --- | --- | --- | --- | --- | --- | --- | --- |
| **Mutation** | **Gly88Cys** | | | | **Ala90Val** | | | | **Ser91Pro** | | | **Asp95His** | | **Asp95Tyr** | | **Asp95Gly** | | | | **Asp95Ala** |
| **Moxifloxacin** | 32 | 8 | 32 | 32 | 8 | 16 | 8 | 8 | 8 | 8 | 8 | 32 | 16 | 16 | 16 | 16 | 16 | 16 | 16 | 8 |
| **Levofloxacin** | 32 | 16 | 32 | 32 | 8 | 8 | 8 | 8 | 8 | 8 | 8 | 16 | 16 | 16 | 16 | 16 | 16 | 16 | 16 | 8 |
| **Gatifloxacin** | 8 | 8 | 8 | 8 | 4 | 8 | 4 | 4 | 4 | 8 | 4 | 8 | 8 | 8 | 8 | 8 | 8 | 8 | 8 | 4 |
| **Ciprofloxacin** | 32 | 16 | 16 | 16 | 16 | 16 | 8 | 8 | 8 | 16 | 8 | 32 | 16 | 16 | 16 | 16 | 32 | 16 | 16 | 16 |

| **Levofloxacin** | 0.385 |  |  |
| --- | --- | --- | --- |
| **Gatifloxacin** | ＜0.001 | ＜0.001 |  |
| **Moxifloxacin** | 0.629 | 0.428 | ＜0.001 |
|  | **Ciprofloxacin** | **Levofloxacin** | **Gatifloxacin** |
